# Supplementary material for: A member of the tryptophan-rich protein family is required for efficient sequestration of Plasmodium berghei schizonts
Source: PLoS Pathog. 2022 Sep 20;18(9):e1010846. doi: 10.1371/journal.ppat.1010846 (PMC9524624; doi:10.1371/journal.ppat.1010846)
Supplement: S2 Table — (PDF) [file ppat.1010846.s002.pdf]

S2 Table: Primers used in this study

#	Direction	Purpose	Restriction enzyme	Sequence
1	fwd	*IPIS3* into B3d+mCherry	SacII	ACTCCGCGGTAATAACGTAAATCCAATTCC
2	rev	*IPIS3* into B3d+mCherry	XbaI	CCATCTAGATATAAATATCGAGTTTCGAAGATATGGGTTC
3	fwd	PBANKA_140070 into B3d+mCherry	SacII	CCTCCGCGGTTTAGATTTAATAATTAATAAGTGCC
4	rev	PBANKA_140070 into B3d+mCherry	XbaI	CGGTCTAGACGAAGAATTTATATGAATATAATACAATACACCC
5	fwd	*IPIS2* into B3d+mCherry	SacII	ACTCCGCGGCTTTTTATTCATGC
6	rev	*IPIS2* into B3d+mCherry	XbaI	CAGTCTAGATTGGAATTTTATATATC
7	fwd	5' integration of IPIS2-mCherry		TTA GAA CAT TGT TAG AAC ATA CCT CGT TGC
8	rev	5' integration of tagging constructs		
9	fwd	3' integration of tagging construts		TAATACGACTCACTATAGGG
10	rev	3' integration of IPIS2-mCherry		CGGGTTTGGTTATCCTTATTTTGACAAAAT
11	fwd	5' integration of IPIS3-mCherry		TATCCCTCATAATGGGGTTATGAATTTTTTC
12	rev	3' integration of IPIS3-mCherry		TAAAAGAAAAATAGCAGCAAATAACGTAATTATC
13	fwd	5' integration of PBANKA_140070-mCherry		TTT GAA AAA ATA TAT AGC ACG CGA TAT TCC
14	rev	3' integration of PBANKA_140070-mCherry		TGAAATAACCACTAGTGAACAATATAAAATC
15	fwd	*IPIS2* 5' UTR	SacII	ATCCCGCGGAGAACATTGTTAGAACATACCTCGTTG
16	rvs	*IPIS2* 5' UTR	NotI	GGATGCGGCCGCTTTGAAGATATTTTATAATATTAATGATAAAATTG
17	fwd	*IPIS2* 3' UTR	XhoI	ATCACTCGAGATTCTATAAAAATTATTAACTAATC
18	rvs	*IPIS2* 3' UTR	KpnI	AACGGTACCGAAACATGCACTTTCTAATATTATTC
19	fwd	*IPIS3* 5' UTR	SacII	ATCCCGCGGTTTACATTAATGTGTGAGATTTTGG
20	rvs	*IPIS3* 5' UTR	NotI	GTATGCGGCCGCAGTGATTTACAAAGATCAAAAATAAAAATAAG
21	fwd	*IPIS3* 3' UTR	XhoI	CATCCTCGAGTGATTCTATATTACATAATCATATTAATC
22	rvs	*IPIS3* 3' UTR	KpnI	TCCGGTACCATAAACGGATTACATAAAATGATTC
23	fwd	*ipis2*-[GFP-Luc;mCherry] integration check		GTGTGTTCCGCATAATGCAAAGTTTTC
24	rvs	*ipis2*-[GFP-Luc;mCherry] integration check		GGATTTAAATGTAACATTGTTCTATCGATGC
25	fwd	*ipis3*-[GFP-Luc;mCherry] integration check		CAATCAATACTTCAAATATACGTTTCCAC
26	rvs	*ipis3*-[GFP-Luc;mCherry] integration check		ATATTAATTACATTTTATTATTGGTTCAATGC
27	fwd	*IPIS2* wild type		TGTATTGTGGCAAATAGCTCAAACTTCTTGAG
28	rvs	*IPIS2* wild type		CAAAGTTTGTTTCTCAGCTTTCCATCGTCC
29	fwd	*IPIS3* wild type		GAATCGATTTCCGTAGGTGCTAGCGC
30	rvs	*IPIS3* wild type		GAACTTGTATCGGTATCTTCTTTCTTTTCATCGG
31	fwd	*Ibis2*-[GFP-Luc;PyrS] Recycle screen – mCherry excised		GATGGAAGCGTTCAACTAGCAGACC
32	rvs	*Ibis2*-[GFP-Luc;PyrS] Recycle screen – mCherry excised		GGATTTAAATGTAACATTGTTCTATCGATGC
33	fwd	*ipis3*-[GFP-Luc;PyrS] Recycle screen – mCherry excised		GATGGAAGCGTTCAACTAGCAGACC
34	rvs	*ipis3*-[GFP-Luc;PyrS] Recycle screen – mCherry excised		CAATCAATACTTCAAATATACGTTTCCAC

35	fwd	*ipis3*-[GFP-Luc]:IPIS2-mCherry - 5' integration	TTA GAA CAT TGT TAG AAC ATA CCT CGT TGC
36	rvs	*ipis3*-[GFP-Luc]:IPIS2-mCherry - 5' integration	GATCCTTACTTGTACAGC
37	fwd	*ipis3*-[GFP-Luc]:IPIS2-mCherry - 3' integration	CTTATCGATACCGTCGACCTC
38	rvs	*ipis3*-[GFP-Luc]:IPIS2-mCherry - 3' integration	CGGGTTTGGTTATCCTTATTTTGACAAAAT
39	fwd	*ipis2*-[GFP-Luc]:IPIS3-mCherry - 5’ integration	TATCCCTCATAATGGGGTTATGAATTTTTTC
40	rvs	*ipis2*-[GFP-Luc]:IPIS3-mCherry - 5’ integration	GATCCTTACTTGTACAGC
41	fwd	*ipis2*-[GFP-Luc]:IPIS3-mCherry - 3’ integration	CTTATCGATACCGTCGACCTC
42	rvs	*ipis2*-[GFP-Luc]:IPIS3-mCherry - 3' integration	TAAAAGAAAAATAGCAGCAAATAACGTAATTATC
43	fwd	T3 promoter sequencing	ATTAACCCTCACTAAAG
44	rvs	mCherry sequencing	TCCATGTGCACCTTGAA
45	rvs	*Pb*DHFR sequencing	TAGATAAAGAACACCTTGTTTATTTTTCCCC
46	fwd	*Pb* 18S qPcR	AAGCATTAAATAAAGCGAATACATCCTTAC
47	rvs	*Pb* 18S qPCR	GGAGATTGGTTTTGACGTTTATGTG
48	fwd	mGAPDH qPCR	CGTCCCGTAGACAAAATGGT
49	rvs	mGAPDH qPCR	TTGATGGCAACAATCTCCAC
