# Supplementary figures and images for: A member of the tryptophan-rich protein family is required for efficient sequestration of Plasmodium berghei schizonts
Source: PLoS Pathog. 2022 Sep 20;18(9):e1010846. doi: 10.1371/journal.ppat.1010846 (PMC9524624; doi:10.1371/journal.ppat.1010846)

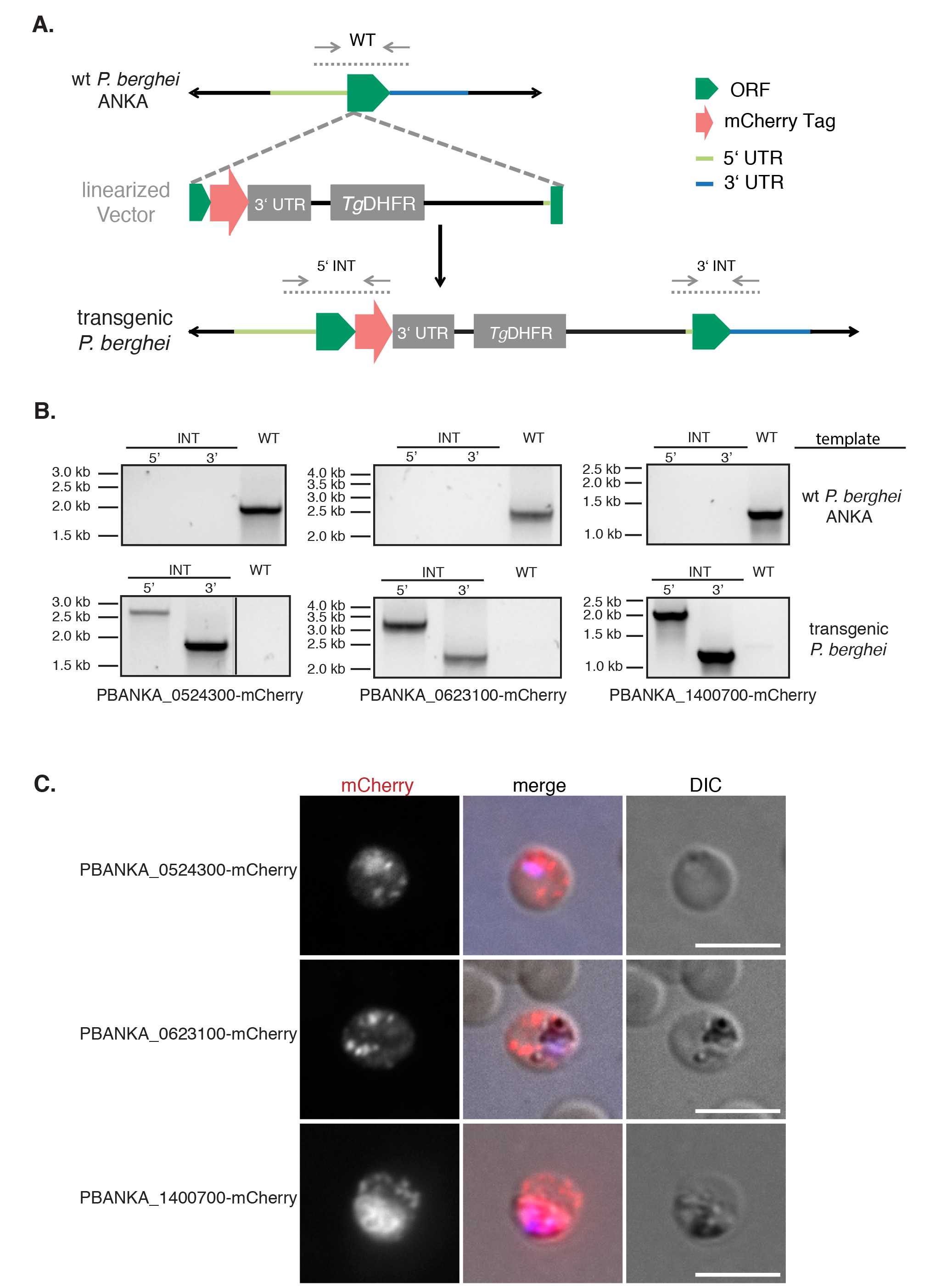

Supplement: S1 Fig — (A) Schematic of the endogenous C-terminal tagging with mCherry. Constructs were integrated into parasite genomic DNA through a single crossover homologous recombination event. Primers used for the genotyping are indicated as grey arrows. (B) Diagnostic PCR with gDNA of P. berghei wild-type parasites (top panel) and transgenic parasites obtained after transfection (bottom panel) confirmed the integration of the linearized vector into the genome. (C) PBANKA_0524300-mCherry, PBANKA_0623100-mCherry, and PBANKA_1400700-mCherry are detected in punctate structures in the cytoplasm of fixed infected erythrocytes. (TIF) [file ppat.1010846.s003.tif]

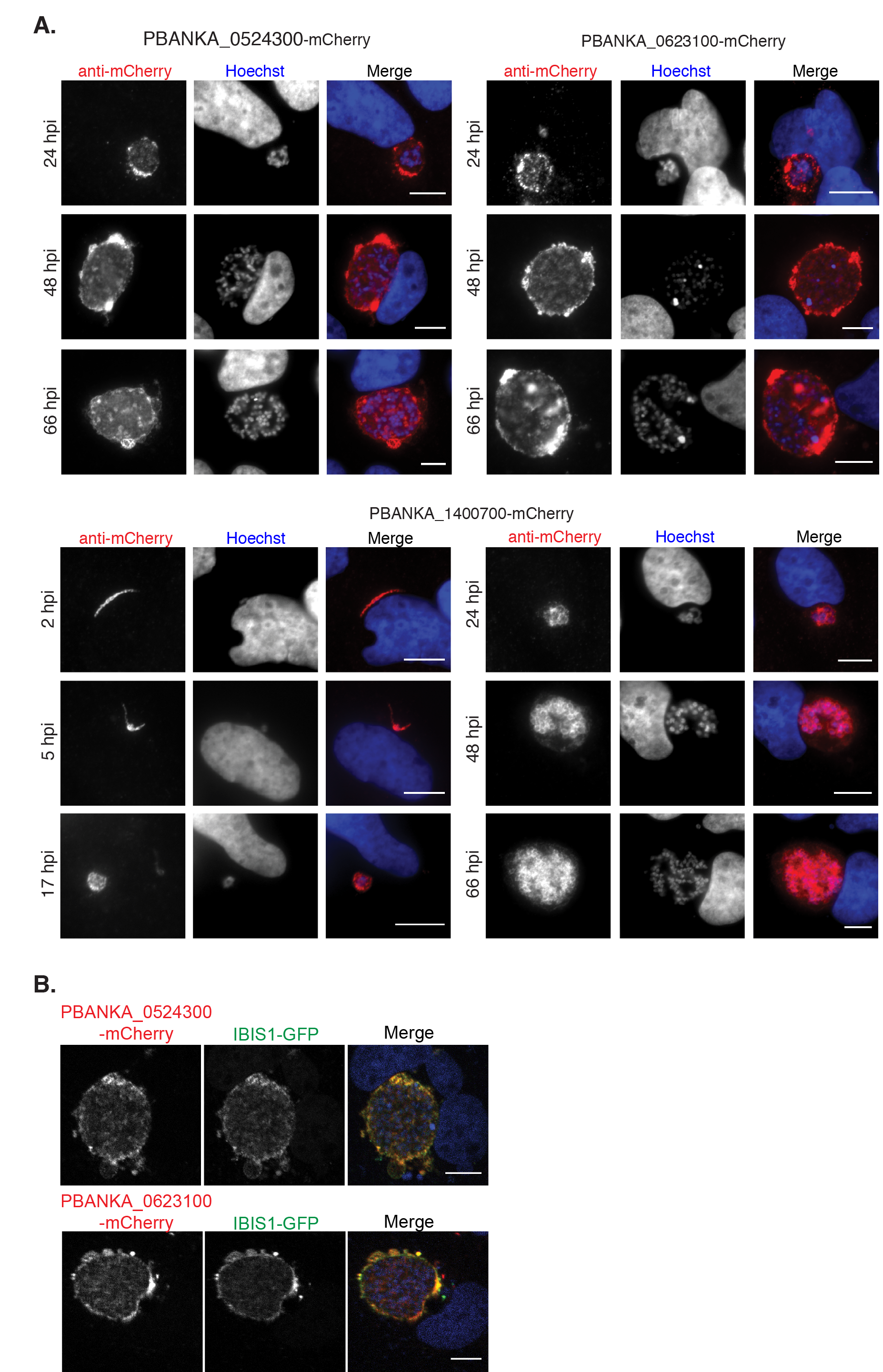

Supplement: S2 Fig — (A) Hepatoma cells were infected with transgenic parasites expressing the indicated proteins fused to the fluorescent protein mCherry. After fixation at the time points indicated, the infected cells were labelled with an anti-RFP antibody to amplify the mCherry signal. Scale bars, 10 μm. (B) P. berghei expressing both GFP- and mCherry-tagged proteins following cross fertilization were imaged by confocal microscopy 48 hours after infection of hepatoma cells. Scale bars, 10 μm (TIF) [file ppat.1010846.s004.tif]

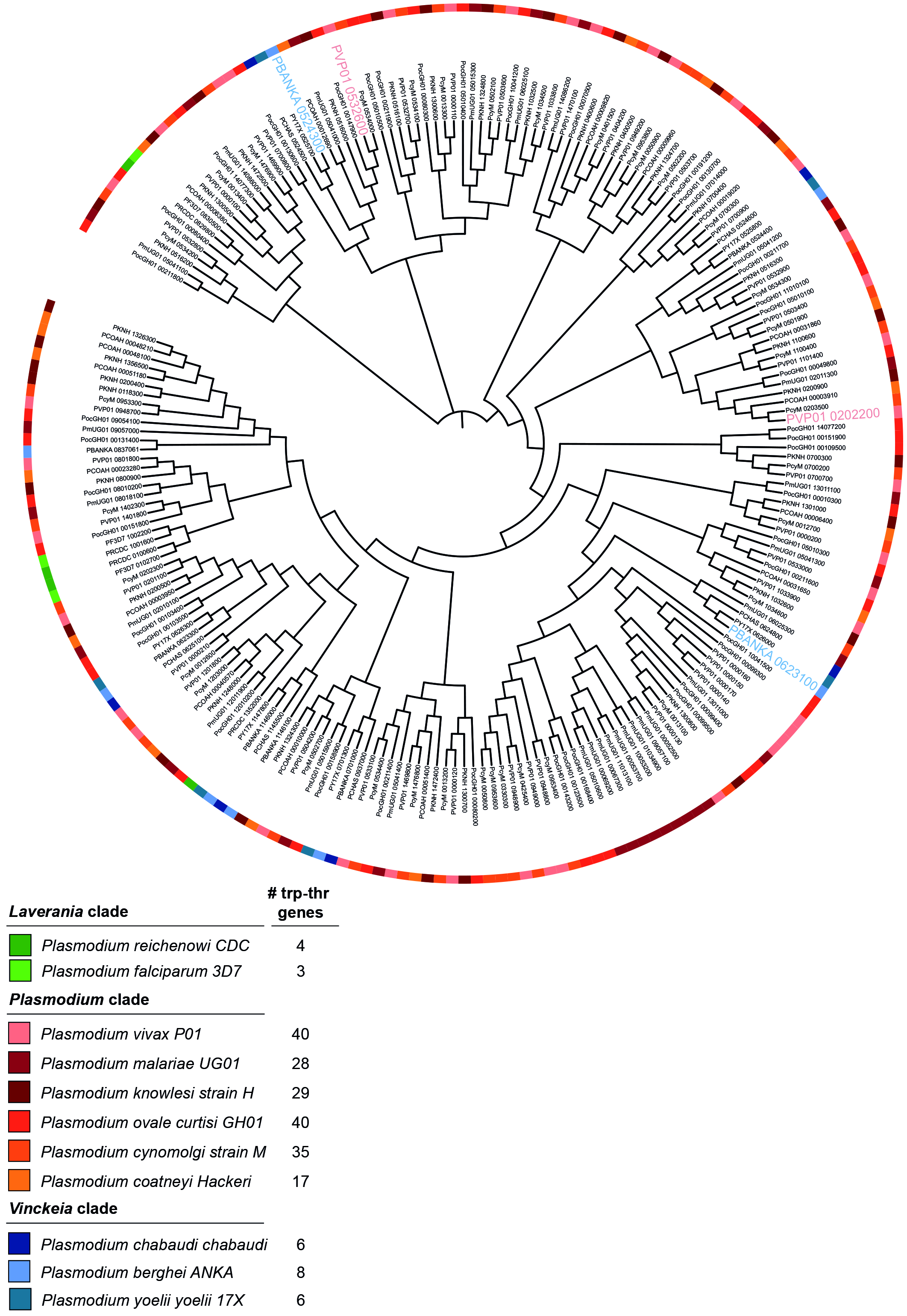

Supplement: S3 Fig — Neighbor-joining tree representing the phylogeny of TryThr domain containing proteins based on multiple sequence alignment of amino acid sequences using MUSCLE. The tree was visualized and annotated using iTOL. For better representation branch length were ignored. The color strip indicates the Plasmodium strains from which the proteins originate. Proteins used in this study are highlighted in blue for Plasmodium berghei and in light red for Plasmodium vivax, respectively. (TIF) [file ppat.1010846.s005.tif]

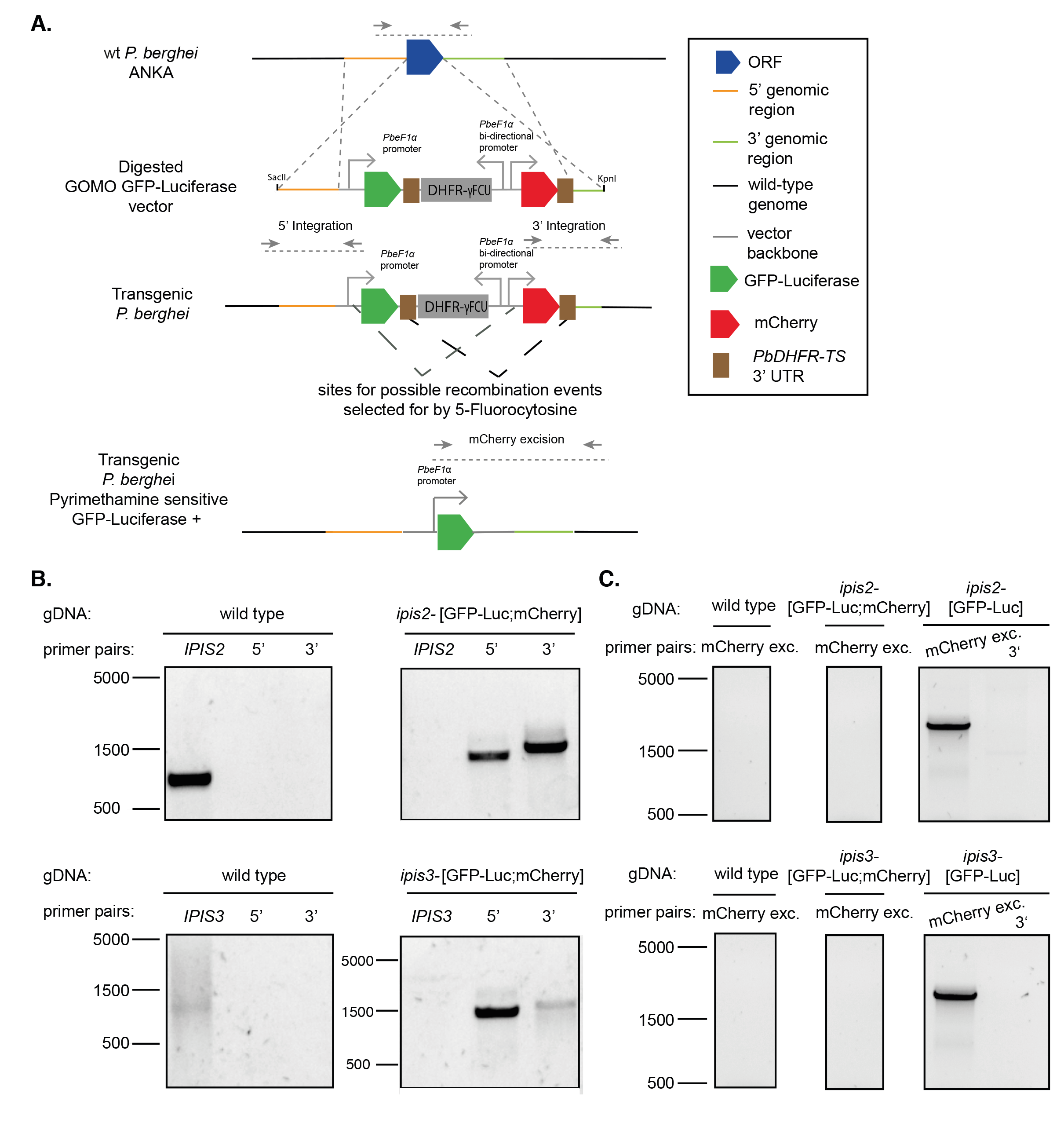

Supplement: S4 Fig — (A) Homology regions (labeled as the 5’ and 3’ genomic regions) flanking the gene of interest were cloned into the GOMO GFP-Luciferase vector. The resulting parasites lack either IPIS2 or IPIS3, express both mCherry and GFP-luciferase, and contain a DHFR-γFCU drug resistance cassette. (B) Successful integration of the construct was verified by PCR amplification of the products indicated in grey in panel A. Produ cts were sequenced to confirm the genomic editing. (C) Excision of the mCherry and drug resistance expression cassette from the recycled lines was confirmed by the presence of product using the mCherry excision (mCherry exc) and absence of the 3’ integration (3’) product. (TIF) [file ppat.1010846.s006.tif]

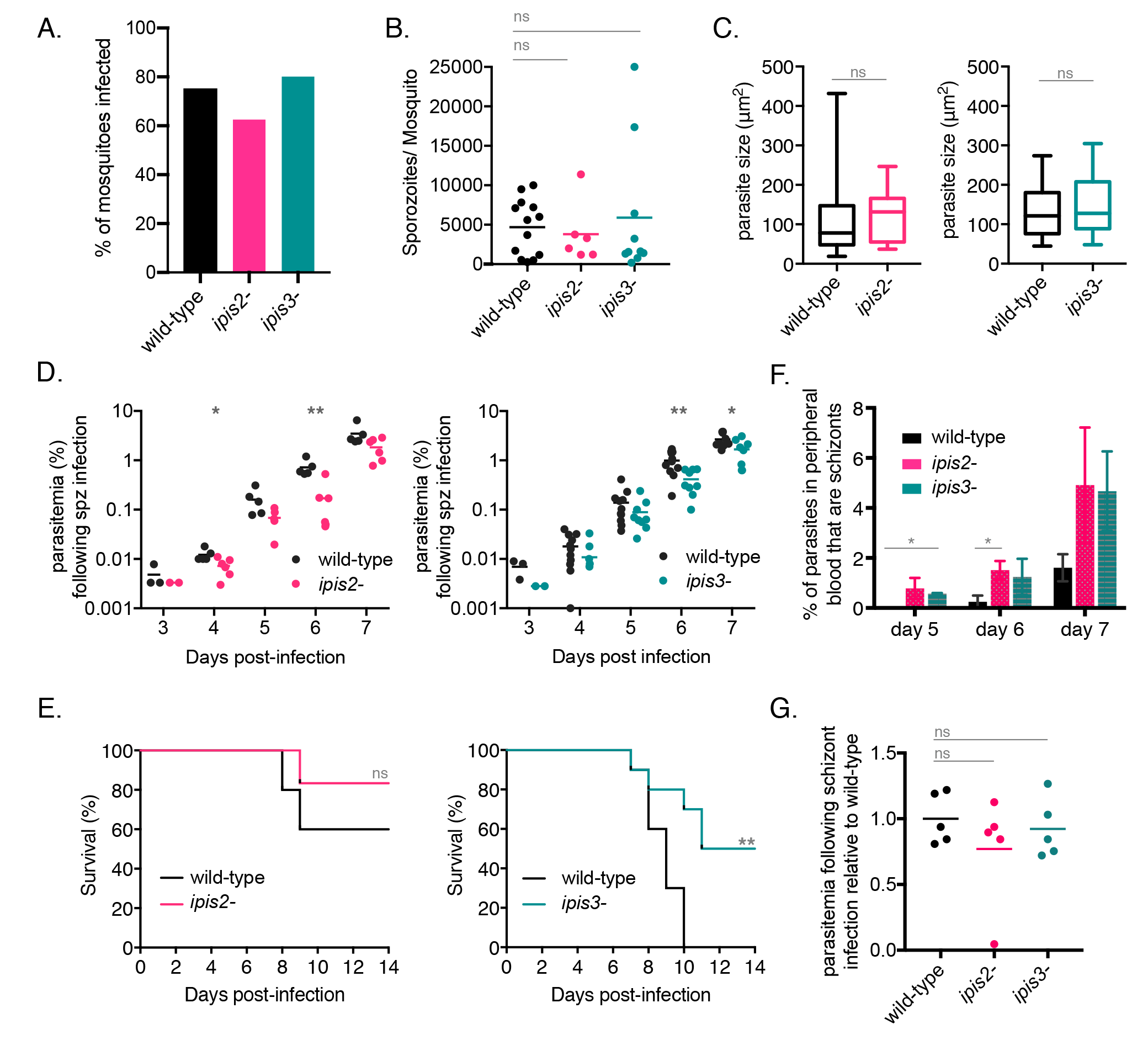

Supplement: S5 Fig — (A) Midguts of mosquitoes were harvested 14 days after an infectious blood meal and microscopy was used to determine the presence or absence of oocysts. (B) The number of sporozoites per mosquito were calculated at least 17 days following infected blood meal. (C) Liver stage ipis2- and ipis3- parasites grow similarly to wild-type parasites in vitro. HepG2 cells infected with either wild-type, ibis2-[GFP-Luc;mCherry], or ibis3-[GFP-Luc;mCherry] sporozoites were fixed 48 hours after infection, and the parasites were stained with antibodies against PbHSP70. Exo-erythrocytic forms were imaged with the fluorescence microscope, and their size was determined using FIJI. ns, not significant, unpaired t-test. (D) Parasitemia in the blood of mice following infection with 1000 sporozoites was quantified by microscopy from Giemsa-stained blood smears. *, P < 0.05, **, P < 0.01, unpaired t-test. (E) ipis2- and ipis3- are less pathogenic in mice than wild-type P. berghei. Survival of mice after infection with 1000 sporozoite. n = 5 (ipis2-), n = 10 (ipis3-). ns, not significant **, P < 0.01, log-rank test. (F) Mice were infected with 5000–10000 infected red blood cells. Parasites in the infected mice were quantified and classified by stage from Giemsa-stained smears of peripheral blood at the indicated days following infection. *, P < 0.05, unpaired t-test. (G) Mice were infected with equal numbers of schizonts isolated from overnight cultures. Parasites in the infected mice were quantified from Giemsa-stained smears of peripheral blood 18–20 hours after infection. ns, not significant, unpaired t-test. (TIF) [file ppat.1010846.s007.tif]

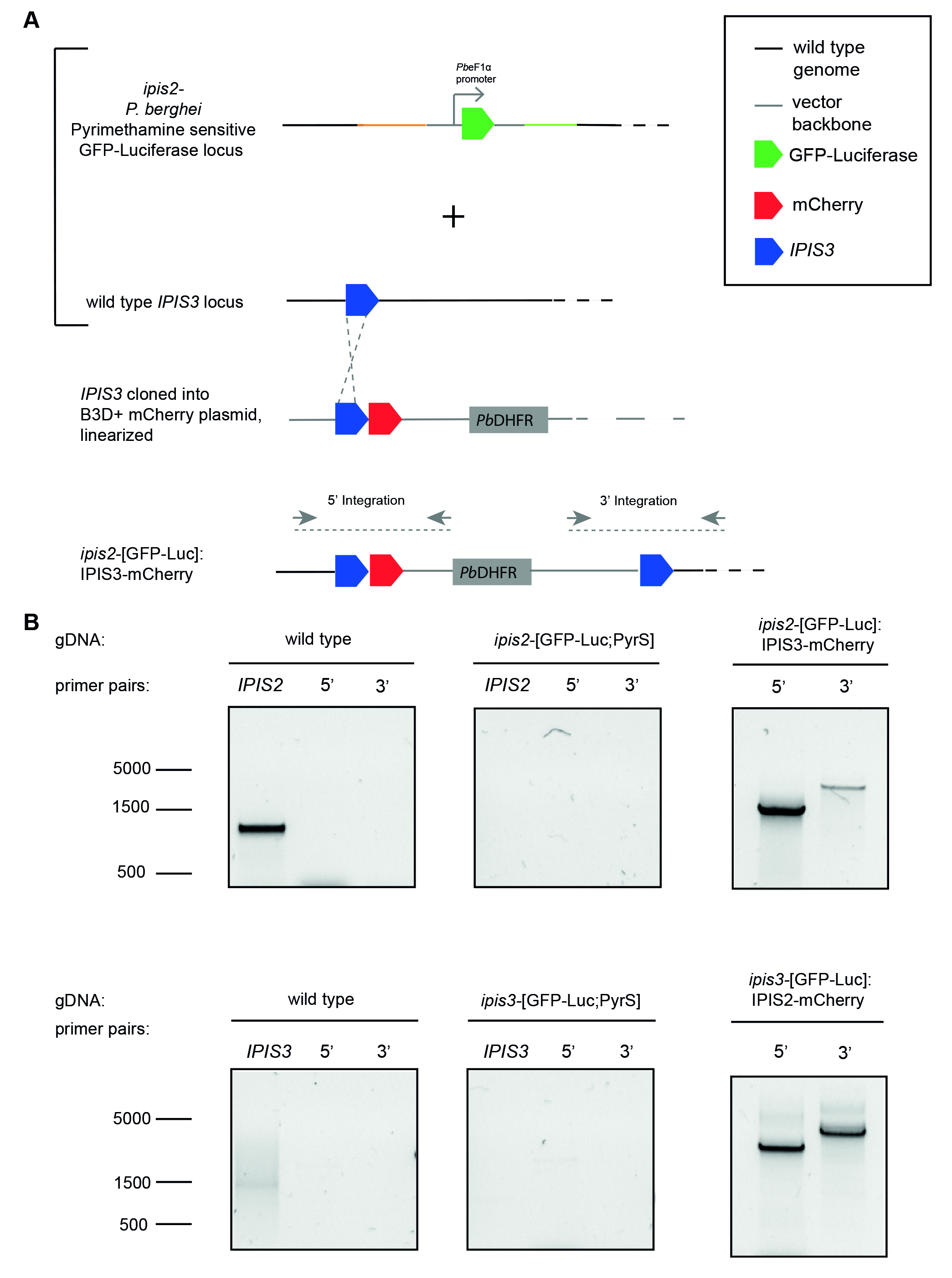

Supplement: S6 Fig — (A) ibis3-[GFP-Luc; PyrS] or ibis2-[GFP-Luc; PyrS] were used as recipient lines for transfection of IPIS2 and IPIS3 B3D+mCherry vectors, respectively. (B) Successful integration of the plasmid was confirmed by PCR. The absence of product for the 5’ and 3’ integration PCRs in the wild type, ipis2-[GFP-Luc;PyrS], and ipis3-[GFPLuc;PyrS] were negative controls. The PCR reactions labeled “IPIS2” or “IPIS3” amplified a region in the IPIS2 or IPIS3 genes, which are absent from the recipient knockout lines. (TIF) [file ppat.1010846.s008.tif]

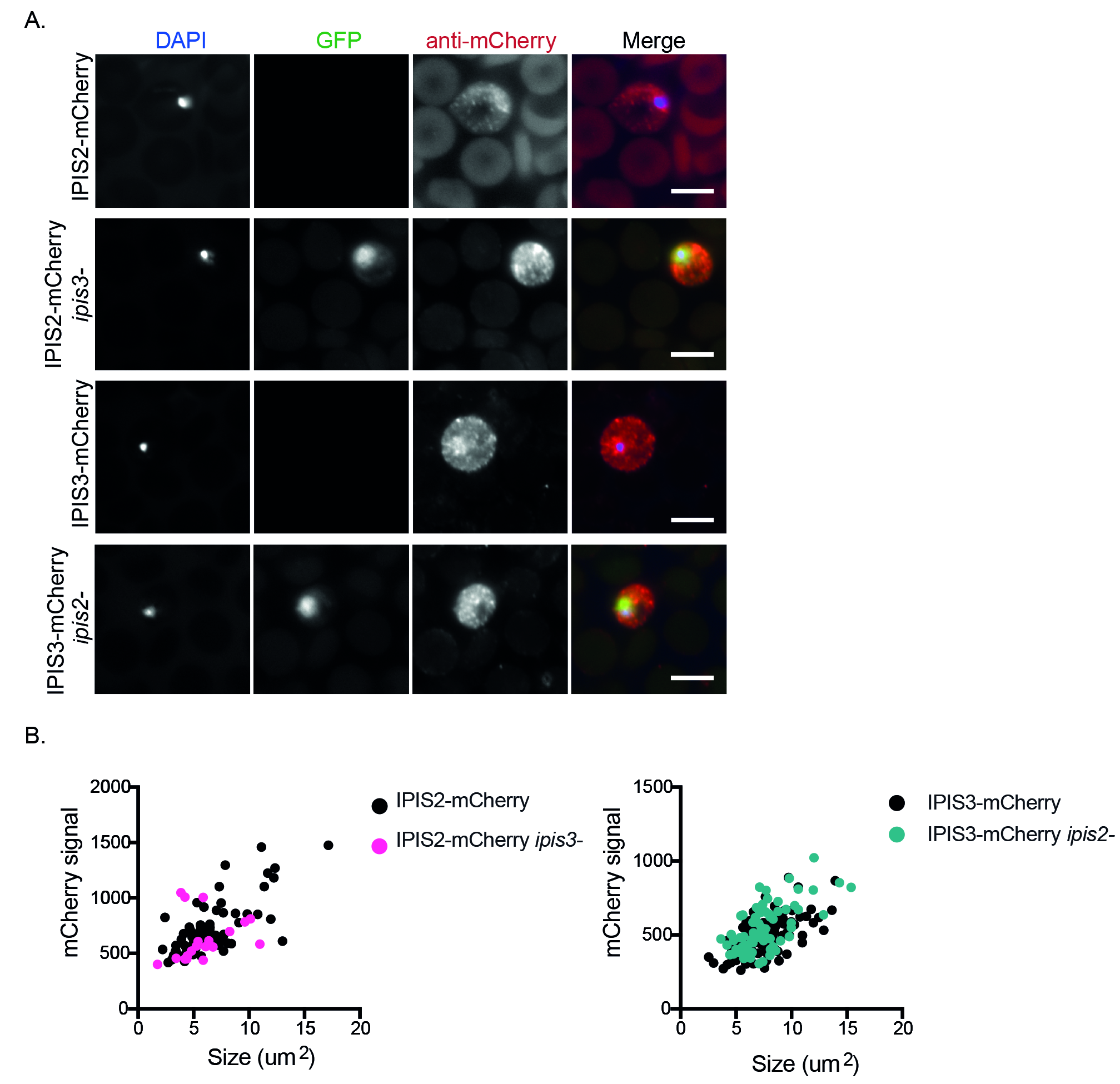

Supplement: S7 Fig — (A) Erythrocytes infected with P. berghei expressing mCherry-tagged proteins in either a wild-type or knock-out genetic background were fixed, labeled with anti-mCherry antibodies and fluorescence microscopy was used to detect the presence of the IBIS via detection of either IPIS2-mCherry or IPIS3-mCherry. DAPI depicts parasite nuclear labelling. GFP is expressed in the parasite cytoplasm exclusively in the knock-out lines. The mCherry signal was amplified by immunofluorescence using antibodies recognizing mCherry. Scale bar = 10 μm. (B) Erythrocyte-associated mCherry signal was quantified in fixed, unstained infected cells. The intensity of the mCherry signal from IPIS2-mCherry and IPIS3-mCherry in both wild-type and knock-out backgrounds was plotted against parasite size. (TIF) [file ppat.1010846.s009.tif]
