# Supplementary material for: A member of the tryptophan-rich protein family is required for efficient sequestration of Plasmodium berghei schizonts
Source: PLoS Pathog. 2022 Sep 20;18(9):e1010846. doi: 10.1371/journal.ppat.1010846 (PMC9524624; doi:10.1371/journal.ppat.1010846)
Supplement: S8 Fig — (PDF) [file ppat.1010846.s010.pdf]

Figure S8: Synthesized sequences of *PvTRAg8* and *PvTRAg2*

PvTRAg8
CATATGGCGGCCGCAAAATGATGGCAACTCAGATTTATAACCCTCCTGGTCGTCAGTTAATGCGT
CGTACACGTGCACCATTACCTATGCCTCCTACAAATAAAAACAATAGCAGTTTTTTAGCAGTATT
GTTAGTGCAATTATGTATTTATTTAGCAGTTCATCAGTACTGTTTAGTAATATTGCACATCCTCCTC
CAAATACTAATAATTTACAGTTTAATGCACCTCATGCAATTGAATATCATCCACGTTTATTGGATAA
AACAGAAGAATGGAAAGATAACGAGTGGAAAAATTGGATGGGTAAATTAGAAGGTGAATGGGTTG
ATTTTAACAACACTATTGAAAAAGAAAAAGGTAAATGGTTAAACGGTAAAGAAAAAGATTGGGACG
AATTTATTCAATATATGGATAACAAATGGATGCATTATCATGATGGTTTAGATGAAGAAGTTAAAAG
TGATATTTTGAAACGTAGCTTAACATTAGATGAAATTGAGTGGAAAGACTGGATTAAAACCGAAGG
AAAAGAATTAATGGAAAAAGATTGGAAAAACTGGATTACTAACAACGAATCATATTTGGATGTTTG
GAGTGTAAAAGAATGGTTAAAATGGAAAAATCAACGTATTGTTACATGGATTATGACTGATTGGAA
ATGTGAAGAAGATGAATATTGGAGTAAATGGGAAGAAAGCTGGGCAAAAAGTCCTAATATGCATG
ATCGTACAAATTGGTTAAATTGGCGTGAACGTTTGAATAAAGAAATGGTTCAATGGAATTCATGGG
TAATGATGAAAGAACAACAAATTCGTGAAAATCGTAGCAACAATTGGTCAAAATGGAAATCAGATA
AACATGTTATGTTTAACTTATGGATGGATACATTTATTAATAAATGGATCAACGAAAAACAATGGTT
TGTTTGGGTTAAAGAACGTAATCAATTACGTTCACGTGGTCGTTATTTAGCATATAAATATCCTTAT
GATGTTCCAGATTATGCATAAACTAGTCTCGAG

PvTRAg2
GCGGCCGCAAAATGGATCAATTTCGTAGTTTACAACATTATATTACTGATCCAAATTTAGTTGCAG
TTTTGAAACAAAAAGCACAAGATCAAATTATGGCATCAATGAATATTTCATTTGCATTTAAAGCATT
ATGTTTTGCTGTTCTTTTTTTCTATGTTGGTGCATTATATGTTAAAAGCAATCAACCAAAAACTGAA
ACAGTTACTAGCCGTAGTAATCCTCATCAAGCAATTGAATATGCAAATCAAGGTCCAAGCCGTGAT
AAAGTTGAAGAATGGAAACGTAATGCATGGACCGATTGGATGGTTCAATTAGATGATGATTGGAA
AGATTTTAACGCACAAATTGAAGAAGAAAAAAAAGCATGGATTGAAGAAAAAGAAGGTGATTGGG
TTATTCTTCTGAAACATTTACAAAATAAATGGCTTCATTTTAACCCAAACTTGGATGCAGAATATCA
AACTGATATGTTAGCAAAATCAGAAACTTGGGATGAACGTCAATGGAAAATGTGGATTAGTACAGA
AGGTAAACAATTATTGGAAATGGATTTAAAAAAATGGTTTACTAACAACGAAATGATTTATTGTAAA
TGGACAATGGATGAATGGAACGAATGGAAAAACGAAAAAATTAAAGAATGGGTTACTTCAGAATG
GAAAGAAAGCGAAGATCAATATTGGAGTAAATATGATGATGCAACAATTCAGACCTTAACTGTTGC
AGAACGTAATCAATGGTTTAAATGGAAAGAACGTATTTATCGTGAAGGAATTGAATGGAAAAACTG
GATTGCAATTAAAGAAAGCAAATTTGTAAACGCAAATTGGAATTCATGGTCAGAATGGAAAAATGA
AAAACGTTTAGAATTTAACGATTGGATTGAAGCTTTTGTTGAAAAATGGATTCGTCAAAAACAATG
GCTTATTTGGACTGATGAACGTAAAAACTTTGCAAATCGTCAAAAAGCAGCACCAGGTGGTGTTG
CAGCAGCACCTGGTGTTTTTGCACCTCGTCCAGCATTTGGTGCACCATCAGGTTTTGCTCCTCGT
CCAGGTTTTGCAGCACCTAGTCAACCTCCACGTTATAGCTTTGCAGCAGCATCAGGTTATGTTGC
ACCATCAGCAACAAGTGAAGCTGCACCTGCAACTTCAGAAGCACCAGCATCAGCAGAAGCAACT
ACAGCATTAAGTAGCGAAACTACTACACCAGTTAATCCTGAAGAAACAGCAGCATCACCAGAAGC
AGCAACCCCTGTTAACCCTGAAGAAACAGCTGCATCAAGTGAAACAACTACAGTTAATCCAGAAG
CAACCCCAGTTAACCCAGAAGCTCCTGTTGCAGAACCTGAAAAAAAAGAAGAAGAACCAGCAGCA
GAACCATTATTGGCAATTGAACCTGCACAAACCGAACCTGCAGCATTAGAAGCTGCACCATCAAC
TAGCGCATATCCTTATGATGTTCCAGATTATGCATAAACTAGT
